# Neural Correlates of Visuospatial Attention to Unseen Stimuli in Hemianopic Patients. A Steady-State Visual Evoked Potential Study

**DOI:** 10.3389/fpsyg.2019.00198

**Published:** 2019-02-06

**Authors:** Javier Sanchez-Lopez, Silvia Savazzi, Caterina Annalaura Pedersini, Nicolò Cardobi, Carlo Alberto Marzi

**Affiliations:** ^1^Psychology and Physiology Section, Department of Neuroscience, Biomedicine and Movement Sciences, University of Verona, Verona, Italy; ^2^Perception and Awareness (PandA) Laboratory, Department of Neuroscience, Biomedicine, and Movement Sciences, University of Verona, Verona, Italy; ^3^National Institute of Neuroscience, Verona, Italy

**Keywords:** hemianopia, attention, visual awareness, blindsight, steady-state VEP

## Abstract

The relationship between attention and awareness is a topic of great interest in cognitive neuroscience. Some studies in healthy participants and hemianopic patients have shown dissociation between these two processes. In contrast, others confirmed the classic notion that the two processes are mutually exclusive. To try and cast further light on this fascinating dilemma, in the present study we have investigated the neural mechanisms of visual spatial attention when perceptual awareness is totally lacking. To do that, we monitored with steady-state visual evoked potentials (SSVEPs) the neurophysiological correlates of endogenous spatial attention to unseen stimuli presented to the blind field of hemianopic patients. Behaviourally, stimulus detection (a brief change in the orientation of a gabor grating) was absent in the blind hemifield while in the sighted field there was a lower, but non-significant, performance in hit rate with respect to a healthy control group. Importantly, however, in both blind and sighted hemifield of hemianopics (as well as in healthy participants) SSVEP recordings showed an attentional effect with higher frequency power in the attended than unattended condition. The scalp distribution of this effect was broadly in keeping with the location of the dorsal system of endogenous spatial attention. In conclusion, the present results provide evidence that the neural correlates of spatial attention are present regardless of visual awareness and this is in accord with the general hypothesis of a possible dissociation between attention and awareness.

## Introduction

Homonymous hemianopia is a visual defect characterized by complete or partial blindness in the hemifield of both eyes contralateral to a lesion of the central visual system (see Bouwmeester et al., 2007). In case of a partial lesion of the optic radiation the visual field defect is usually limited to the contralateral upper or lower quadrant. Importantly, some hemianopic patients have been found to present “blindsight” i.e., unconscious visually triggered behavior (Poppel et al., 1973; Weiskrantz et al., 1974). Following the discovery of this intriguing phenomenon hemianopic patients have become a fundamental source of information on the neural mechanisms of awareness by studying the effects of damage of specific brain areas (Weiskrantz, 2004). This endeavor is clearly impossible in healthy humans.

Many studies have found a dissociation between attention and perceptual awareness in healthy participants (McCormick, 1997; Ivanoff and Klein, 2003; Lu et al., 2012; Block, 2014; Herreros et al., 2017) and a few in hemianopic patients with blindsight (Kentridge et al., 1999, 2004). An important question is what kind of attention might operate without awareness: It has been suggested that this occurs with endogenous rather than exogenous attention (but see Chica et al., 2012 for a different opinion). In hemianopia it has been found that endogenous orientation of spatial attention facilitates performance (mainly reaction time-RT) even in absence of visual awareness and this has led to the conclusion that there exists a dissociation between this kind of spatial attention and perceptual awareness (Kentridge, 2011). How could endogenous spatial attention operate without awareness? In principle, if its mechanisms are similar to those operating consciously they should involve cortical areas such as the frontal eye fields (FEF) and the intraparietal sulcus/superior parietal lobe (IPS/SPL) that constitute the normal dorsal attention system network (Corbetta and Shulman, 2002; Corbetta et al., 2008) and exert top-down influences on visual areas during spatial orienting of attention (Hopfinger et al., 2000; Bressler et al., 2008). However, although the effects of visual spatial attention on behavioral performance to unseen stimuli have been clearly demonstrated, to our knowledge, no studies have been conducted to investigate their neurophysiological correlates. Thus, we still do not know whether attention operating independently from awareness has similar neural bases as those subserving conscious attention. Shedding light on this problem represents the aim of the present study.

Recently, we demonstrated the reliability and effectiveness of steady-state visual evoked potentials (SSVEP) in the study of unconscious passive visual processing in hemianopic patients (Sanchez-Lopez et al., 2017). SSVEPs are repetitive visual stimuli presented at a high rate, usually between 10 and 20 Hz that elicit an entrainment of the brain electrical activity at the same frequency of the driving stimulus and its harmonics. SSVEPs reflect high propagation properties, are less influenced by artifacts, require less time for data acquisition, have a higher signal-to-noise ratio (Di Russo et al., 2003; Vialatte et al., 2010), and can be measured in time and preferably in the frequency domain (Vialatte et al., 2010; Schomer and Lopes da Silva, 2011).

Morgan et al. (1996) have recorded SSVEPs from participants who were cued to attend to visual stimuli presented to one hemifield and to ignore the concurrent stimulation on the opposite hemifield. They found that the amplitude of the frequency SSVEP was significantly enlarged when attention was focused on the attended location and was larger over occipital and temporal scalp areas. This finding provided the basis for the study of the neural mechanisms of selective attention to multi-element visual displays (for a review see Vialatte et al., 2010; Andersen et al., 2011) in healthy participants. However, as mentioned above, it is still to be understood whether the neural mechanisms of attention operating in the absence of awareness are similar to those during awareness. This is an important query that we purported to tackle in the present study whose rationale is straightforward: If the mechanisms of attention are similar independently from stimulus awareness then we would expect a qualitatively similar enhancement of the SSVEP response to stimuli in the attended intact or the blind field of hemianopic patients. On the contrary, if attention operating without awareness relies on different neural bases this should show up as a differential response in the blind versus intact field of hemianopic patients or healthy participants.

## Materials and Methods

### Participants

#### Patients

Five hemianopic patients (3 females and 2 males; mean age = 54.4 years, SD = 7.3) with post-chiasmatic lesions participated in the study. Two of them had quadrantanopia (one upper and the other lower). Three patients had right and the other two left hemisphere damage, see Table 1. Inclusion criteria were: Diagnosis of hemianopia made at least three months before testing, availability of visual campimetry and structural MRI documenting the site and extension of the brain damage. Exclusion criteria included pre-existing neurologic or psychiatric disorders, drugs or alcohol addiction, cognitive impairments evidenced by a score equal or less than 24 in the Mini Mental State Examination (Folstein et al., 1975), and presence of hemineglect as assessed with a neuropsychological battery including: Line Bisection (Schenkenberg et al., 1980), Diller letter H cancelation (Diller et al., 1974), and Bell Cancelation (Gauthier et al., 1989). Additionally, patients were evaluated with the Visual Function Questionnaire (VFQ25), in order to assess subjective impressions on their visual abilities in everyday life (Mangione et al., 2001). All patients were right handed and had normal or corrected to normal visual acuity. A brief description of patients’ lesion location and campimetry can be found in Table 1; for a detailed description of the patients see also Sanchez-Lopez et al., 2017.

**Table 1:** Patients’ clinical description.

Patient (age/gender)	Lesion/Visual Deficit	Campimetry (left eye/right eye)
FB (49/F) Right hemisphere lesion	*Neuroradiological description:* Lesion involving the temporal, parietal and occipital lobe. In the latter, the lesion includes the superior and part of the middle occipital gyri with interruption of the optic radiation. *Specific structures affected:* Anterior intraparietal sulcus, visual area V5, inferior parietal lobule, somatosensory cortex, primary auditory cortex, parietal operculum and insula in the right hemisphere. *Visual defect:* Left lateral homonymous hemianopia.	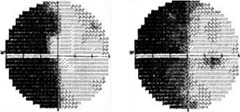
LF (50/F) Right hemisphere lesion	*Neuroradiological description:* Ischemic lesion that involves the cortex of the anterior half of calcarine fissure to the origin of parieto-occipital fissure. *Specific structures affected:* Small internal portion of V1 and V2. *Visual defect:* Upper left homonymous quadrantanopia.	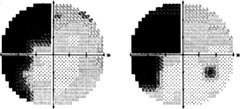
LC (66/M) Right hemisphere lesion	*Neuroradiological description:* Temporal and parietal lesion, with posterior extension to the white matter of occipital lobe, involving the lateral part of optic radiation. *Specific structures affected:* Middle, inferior and a small portion of the superior temporal gyrus. Visual area V5 and inferior parietal lobe. *Visual defect:* Left lateral homonymous hemianopia.	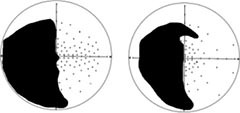
GA (60/M) Left hemisphere lesion	*Neuroradiological description:* Ischemic lesion involving parietal and occipital lobe. In the latter the lesion involves the superior, middle, inferior and descending occipital gyri, cuneus, pole and the posterior part of optic radiation, with relative sparing of the lingual and fusiform gyri. *Specific structures affected:* Small portion of anterior intraparietal sulcus and all visual areas (V1,V2,V3,V4 and V5). *Visual defect:* Lower right homonymous quadrantanopia.	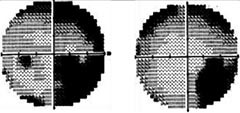
SL (47/F) Left hemisphere lesion	*Neuroradiological description:* Lesion involving the median para-sagittal portion of the occipital lobe. The lesion includes the lingual gyrus, with peri-calcarine fissure distribution. *Specific structures affected:* V1, V2, V3, and V4 visual areas. *Visual defect:* Right lateral homonymous hemianopia.	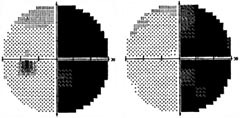


#### Healthy Participants

In addition to hemianopic patients we tested a group of 18 young healthy participants (13 females; mean age = 25.2 years old, SD = 4.0). All of them were right handed and had normal or corrected to normal visual acuity.

Informed consent to take part in the study was obtained from all healthy and hemianopic participants after they had been informed about the procedures and their rights. The study was approved by the Ethics Committee of the European Research Council and of the Verona Azienda Ospedaliera Universitaria Integrata (AOUI). All subjects gave written informed consent in accordance with the Declaration of Helsinki.

### Stimuli

**FIGURE 1:**
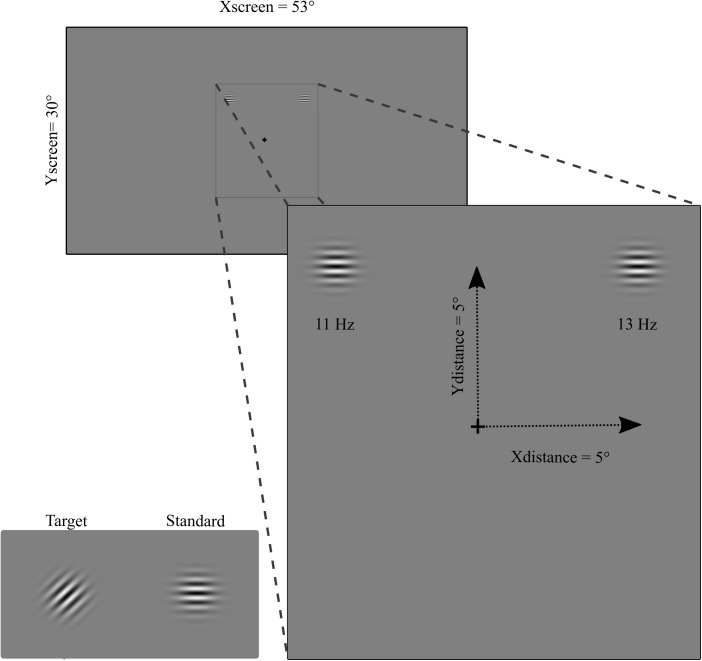
Stimuli (target and standard), flickering frequency (11 Hz and 13 Hz on left and right hemifield, respectively), and eccentricity (for healthy participants).

The visual stimuli consisted of circular black and white horizontal (standard stimulus) and 45° oriented (target stimulus) Gabor gratings. The diameter of the stimuli was 2° of visual angle with a spatial frequency of 4 c/° (see Figure 1). The contrast of the Gabor grating was 0.8 and the background luminance was the same as the mean luminance of the Gabor (17.7 cd/m^2^). Flickering stimulation was obtained by contrast reversal each 90.9 ms (i.e., 11 Hz) and 79.9 ms (i.e., 13 Hz) for left and right hemifields, respectively. Two different frequencies for left and right hemifield were used in order to evaluate simultaneously two-element visual displays i.e., attended and unattended stimuli as done in previous studies recording SSVEP during attention tasks (Morgan et al., 1996; Vialatte et al., 2010). The stimulation was performed by presenting simultaneously two flickering Gabor gratings on a LED video monitor (resolution = 1920 pixels width × 1080 pixels height, and refresh rate = 144 Hz), one to the left and one to the right, in the upper or lower visual field for the patients. The stimulation in the group of healthy participants was performed in both the upper and the lower visual field in a counterbalanced order across subjects. The eccentricity of stimulus presentation for patients depended upon the position of the visual field loss (see below). For healthy participants was *x* = 5° and *y* = 5°.

### SSVEP Stimulation

Participants were comfortably seated at a viewing distance of 57 cm from the screen. The stimuli were binocularly presented and participants were asked to maintain a stable fixation on a central cross during stimulus presentation. Ocular movements were externally controlled through a closed-circuit camera. Constant feedback about their ability to maintain fixation was given to the participants. SSVEP stimulations consisted of 40 blocks each of 18.3 s of simultaneous left and right hemifield stimulation. Brief breaks were intermingled between blocks. At the beginning of each block participants were asked to pay attention, for the entire block, to the left or right hemifield. Attention was alternated left and right hemifield across blocks (20 toward the left and 20 toward the right hemifield). Patients were instructed to press the space bar of the keyboard when the target stimulus, i.e., a brief modification of grating orientation (same duration as the standard stimuli 90.9 and 79.9 ms for left and right hemifield, respectively), appeared in the attended (5 times per block) and to ignore it when appeared in the unattended hemifield (5 times per block). Four hundred target stimuli were pseudo-randomly presented during the session: 200 in the attended side (100 in each left or right hemifield) and 200 in the unattended side (100 in each left or right hemifield). On the right hemifield 4,760 pattern-reversal stimuli were presented for each attended and unattended condition, while in the left hemifield 4,020 stimuli were presented per condition (see Figure 1).

For each participant in the patients’ group the stimulus was positioned in the blind area and in a corresponding area in the intact hemifield on the basis of the results of clinical campimetry, as shown in Table 1 and of a visual mapping test carried out in the lab (for more details see Sanchez-Lopez et al., 2017). In order to check for possible undetected residual vision, at the beginning of the experimental session we evaluated the subjective level of perceptual awareness by moving the stimulus in the blind portion of the visual field and asking the patient whether he/she had some visual sensation. Moreover, at the end of each block, patients were asked if they had ever detected the appearance of the stimulus in the blind field. In both tests all patients reported no visual sensation whatsoever. Patients’ eccentricities of stimulus presentation are shown in Table 2.

**Table 2:** Stimulus position (in degrees), and stimulated visual field for the group of patients. Stimuli were symmetrically positioned in left and right quadrants.

Patient	Stimulus Position (°)		Visual field
	x	y	
FB	13.8°	6.3°	Lower visual field
LF	12.2°	6.4°	Upper visual field
LC	14°	3.3°	Lower visual field
GA	7.3°	2.7°	Lower visual field
SL	4.8°	4.8°	Upper visual field


### EEG Recording

EEG was recorded during the performance of the task. An elastic cap with 59 active electrodes (ActiCap, Brain Products GmbH, Munich Germany) placed according to the 10-10 International System was used. An acquisition system with two BrainAmp amplifiers and the software Recorder 1.2 (Brain Products GmbH, Munich, Germany) was employed. On-line reference was placed on the left mastoid while the right mastoid electrode was used to re-reference the EEG recording offline to the average of the right and left mastoid electrodes. The ground electrode was placed in the AFz electrode position. Horizontal and vertical eye movements were recorded with four electrodes placed at the left and right canthi and above and below the right eye, respectively. The impedance of all electrodes was kept below 5 KΩ. The EEG was recorded at 1000 Hz sampling rate with a time constant of 10 Hz as low cut-off and a high cut-off of 1000 Hz with a 50 Hz notch filter.

### Data Analysis

#### Behavior

The scoring analysis included hit rate (response to target in the attended field), false alarm rate (response to target in the unattended field), reaction times (RT), and d prime (d’). Since patients never responded to blind visual field stimuli, only performance in the sighted visual field was compared with that of a subset of healthy participants randomly sorted to obtain a similar distribution as the patients’ group: 40% performed the task in the upper visual field (half scores obtained from the left hemifield and the other half from the right visual field); 60% of healthy participants performed the task in the lower visual field (2/3 of the scores from the right visual field and 1/3 from the left visual field). Group comparisons were carried out by means of one way ANCOVA using age as covariate independently for each comparison.

#### EEG Pre-processing

The EEG signal was pre-processed offline using EEGLAB toolbox (Delorme and Makeig, 2004), and MATLAB (version R2018a, The MathWorks, Inc., Natick, MA, United States, 2010) scripts. Data pre-processing was carried out for all channels by re-referencing to the average of the right and left mastoid electrodes. Vertical eye movements were corrected by means of Independent Component Analysis (ICA) ocular correction (Makeig et al., 1996). EEG analysis aimed at investigating the rhythmic entrainment produced by the standard stimuli, therefore 2 s overlapped epochs locked to the standard stimuli were obtained from the continuous EEG recording separately for each condition: attended left/blind, unattended left/blind, attended right/sighted, and unattended right/sighted; all segments were band pass filtered from 0.1 to 40 Hz. Baseline correction was performed for each segment by removing the mean value of the signal per channel per trial. Finally, semiautomatic rejection of segments with artifacts was carried out. Clean segments were separately averaged for each condition. Finally, the frequency power, by means of the fast Fourier transformation (FFT), was extracted for each channel of the averaged SSVEP as 2 s segments.

#### SSVEP

For the statistical analysis of the SSVEP responses to the entrainment produced by the standard stimuli, the peak of power at the frequency of stimulation of 11 Hz for left visual field and 13 Hz for right visual field was extracted after the FFT for each condition, electrode and participant. In order to create a single group of patients, the EEG montage of those with left lesion (right hemianopia; *n* = 2) was flipped left to right. For the healthy participants group the EEG montage of the 40% of the participants was flipped left to right as in the patients group. A non-parametric permutation test using 10,000 permutations as implemented in EEGLAB function “statcond” (Delorme and Makeig, 2004) was used.

**Within-subjects** comparisons were performed for each group (healthy participants and patients) separately, by comparing attended versus unattended conditions for each hemifield (left/blind and right/sighted). In order to evaluate hemispheric differences in the effect of attention between contralateral and ipsilateral hemisphere with respect to the visual hemifield, the same statistical analysis was performed for each hemifield in both healthy and patients group.

**Between-subjects** comparisons **(patients versus healthy participants)** concerned the net effect of attention (attended minus unattended condition) for each hemifield.

**FIGURE 2:**
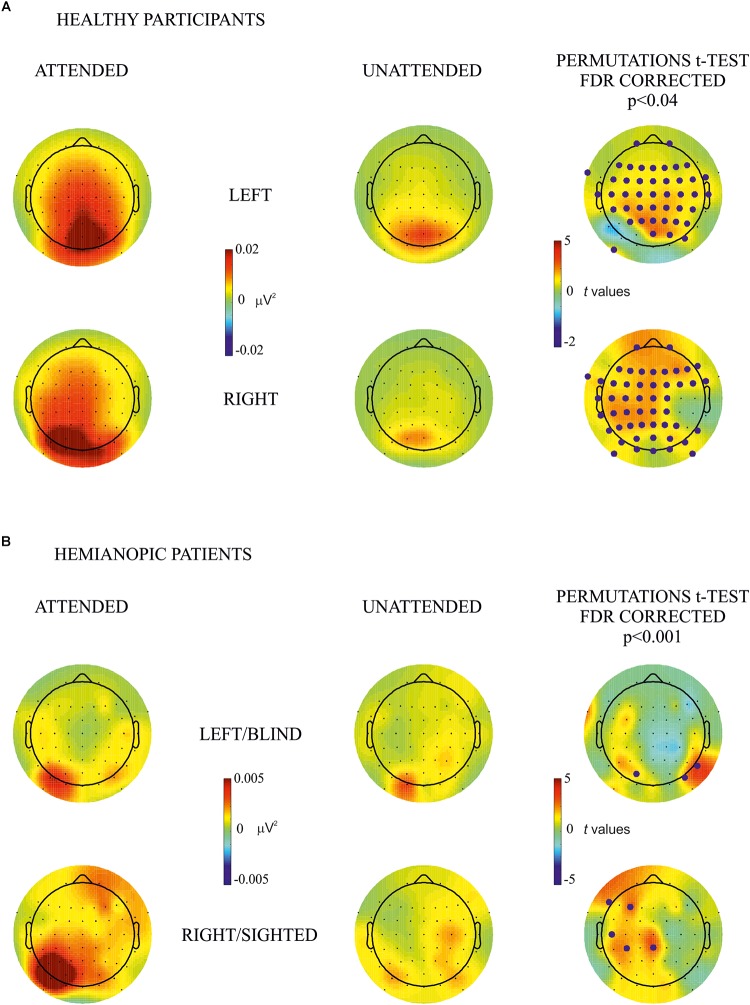
Within-subjects attention effect. Permutation *t*-test comparisons between attended (first column) and unattended (second column) for **(A)** healthy participants and **(B)** hemianopic patients. Topographical maps in columns 1 and 2 represent the power of frequency (μV^2^) for each condition attended and unattended, respectively. Maps in the 3rd column represent *t* values of the comparison between attended and unattended conditions; blue points indicate the electrode sites where the power of frequency in the attended condition was significantly higher than in the unattended condition after the FDR correction calculated separately for each comparison. The maximum *p* value accepted after FDR correction is indicated in the tittle of the 3rd column.

In consideration of the more localized topographical distribution of the SSVEP over posterior electrodes in the group of patients in comparison with healthy participants (see Figure 2), the statistical analyses, where patients were included, were carried out in nine topographical sites separately: frontal left (Fp1, F7, F5, F3, and F1), frontal right (Fp2, F2, F4, F6, and F8), central left (FC5, FC3, FC1, C5, C3, C1, CP5, CP3, and CP1), central right (FC2, FC4, FC6, C2, C4, C6, CP2, CP4, and CP6), temporal left (FT9, FT7, T7, and TP7), temporal right (FT10, FT8, T8, and TP8), posterior left (P7, P5, P3, P1, PO9, PO7, PO3, and O1), posterior right (P2, P4, P6, P8, PO4, PO8, PO10, and O2), and midline (Fz, FCz, Cz, CPz, Pz, POz, and Oz). For the comparison of the effect of attention between contralateral and ipsilateral hemisphere in each hemifield, in the healthy group all electrodes from the contralateral were compared with their counterpart on the ipsilateral hemisphere, and in patients, separately, for each group of lateral electrodes (frontal, central, temporal, and posterior). No midline electrodes were included in this analysis. False discovery rate (FDR) correction was used to adjust *p*-values for multiple comparisons.

## Results

### Behavior

As described in the Methods section, since patients did not respond to the target stimuli in the blind hemifield, only the sighted hemifield was analyzed and compared with its counterpart in the healthy participants group. No differences between groups were observed in any variable tested: percentage of hit rate (*F* < 1; Mean-healthy = 79.2 ± 16.4; Mean-patients = 45.8 ± 30.94), percentage of false alarms (*F* < 1; Mean-healthy = 1.6 ± 2.1; Mean-patients = 15.8 ± 20.6), RT (*F* < 1; Mean-healthy = 494.1 ± 54.6 ms; Mean-patients = 534.6 ± 132.1 ms), and d’ (*F*_(1,20)_ = 1.4; p = 0.2; Mean-healthy = 2.6 ± 1; Mean-patients = 1.4 ± 1.6). The other hemifield of the healthy participants group was analyzed only in terms of descriptive statistics: Mean percentage of hits rate = 80.1 ± 4.5; Mean RT = 479.6 ± 52.5 ms; Mean percentage of false alarms = 2.1 ± 2.5; and Mean d’ = 2.61 ± 0.9.

### SSVEP

**FIGURE 3:**
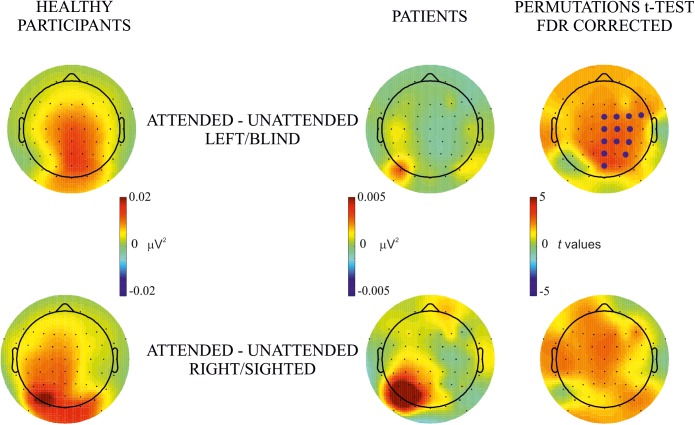
Between-subjects attentional effect. Permutation *t*-test comparisons of the effect of attention (i.e., attended – unattended) between healthy participants and hemianopic patients in blind (upper row) and sighted (lower row) hemifields. Topographical maps in columns 1 and 2 represent the power of frequency (μV^2^) for each condition attended and unattended, respectively. Maps in the 3rd column represent *t* values of the comparison between attended and unattended conditions; blue points indicate the electrode sites where the power of frequency in the attended condition was significantly higher than in the unattended condition after the FDR correction calculated separately for each comparison. The maximum *p* value accepted after FDR correction is indicated in the tittle of the 3rd column.

By visual inspection of the topographic maps it appears that the effect of attention was present for the blind as well as the sighted hemifield of hemianopic patients and was similar, although less pronounced, to that of healthy participants in whom the effect was bilateral while in patients it was more pronounced over the intact hemisphere regardless of hemifield (see Figure 2, 3).

#### Within-Subjects Attentional Effect

The main objective of this analysis was to investigate the difference in frequency power between attended and unattended conditions for stimulus presentation to the same hemifield. Comparisons were carried out for each hemifield separately for both groups. In the **healthy participants group** permutation *t*-test showed a significantly higher power in the attended than the unattended condition in most of bilateral occipital, parietal, temporal and frontal electrode sites (*p* values < 0.04; see Figure 2A). In the **hemianopic patients group** the permutation tests yielded the following statistically reliable differences: For the left/blind hemifield the SSVEP response was higher in the attended than the unattended condition in the posterior groups of electrodes of the intact and lesioned hemispheres (*p* values < 0.001): PO3 (intact hemisphere), PO8, and P8 (lesioned hemisphere). For the right/sighted hemifield there were differences with higher frequency power in the attended than in the unattended condition in the central and frontal group of electrodes over the intact hemisphere (*p* values < 0.001) on CPz, CP3, C5, F3, and F7 (see Figure 2B).

**FIGURE 4:**
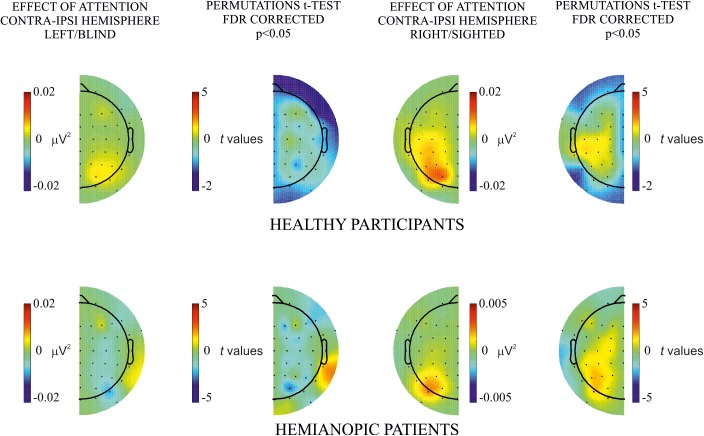
Hemispheric differences of the attentional effect. Permutation *t*-test comparisons of the effect of attention (i.e., attended – unattended) between contralateral and ipsilateral hemisphere for each hemifield, left/blind (left column), and right/sighted (right column), in both healthy (upper row) and patients (lower row) group. No significant differences were observed.

In the analysis of hemispheric differences (contralateral versus ipsilateral) of the effect of attention on hemifield of stimulus presentation, no significant differences were observed in both hemifields (left/blind or right/sighted) of either healthy participants or patients (see Figure 4).

#### Between-Subjects Attention Effect

The purpose of this analysis was to investigate group differences in the effect of attention (attended minus unattended in the same hemifield). Topographical maps of frequency power showed a widespread bilateral effect in **healthy participants** which, in **hemianopic patients** was present mainly over posterior electrodes of the intact hemisphere for both blind and sighted hemifield stimulus presentation, see Figure 3. Permutations tests showed significant differences in posterior, central and frontal groups of electrodes along the midline and in the lesioned hemisphere of patients, while no significant differences were found for the sighted hemifield.

## Discussion and Conclusion

The aim of this study was to investigate the neurophysiological correlates of endogenous spatial attention to unseen stimuli. In the group of **healthy participants** we found significantly larger SSVEP responses in the attended versus unattended condition mainly in occipital but also in frontal, temporal, and parietal sites. These results are in line with previous findings on SSVEP and endogenous sustained attention (Morgan et al., 1996; Andersen and Muller, 2010; Vialatte et al., 2010; Andersen et al., 2011). Importantly, a reliable, albeit less pronounced similar effect, was found in the group of **hemianopic patients**: Following stimulus presentation in the blind hemifield a higher frequency power in the attended than unattended condition was found bilaterally over occipital electrodes. This suggests the influence of top-down attentional mechanisms over spared portions of the striate cortex (Hopfinger et al., 2000; Bressler et al., 2008) and of extrastriate areas. This is a reasonable possibility given that striate (V1) and extrastriate visual areas are considered as responsible for the SSVEP response (see Di Russo et al., 2002; Vialatte et al., 2010; Sanchez-Lopez et al., 2017; Mitka and Riecansky, 2018). In particular, following stimulus presentation to the blind hemifield the contralateral activity observed over PO8 and P8 is likely to originate from extrastriate areas (Di Russo et al., 2002) of the lesioned hemisphere while the activity over PO3 might origin from striate as well as extrastriate areas (Di Russo et al., 2002) of the ipsilateral intact hemisphere. In the sighted hemifield of hemianopics a significant SSVEP difference between attended and unattended stimuli was found over central and frontal electrodes in the intact hemisphere likely originating from extrastriate visual areas (hMT/V5), precuneus, superior, and inferior parietal lobe and middle frontal lobe (Mitka and Riecansky, 2018) of the intact hemisphere, i.e., areas of the dorsal system for endogenous spatial attention (Vossel et al., 2014). One might wonder why we found a significant occipital effect of attention in the blind hemifield and a central and frontal effect in the sighted hemifield. One possibility is that even though in the blind field there was a trend toward an attentional effect in the latter areas it was not larger enough to reach statistical reliability. This might be due to inter subject variability of the lesion in some parietal and temporal areas in different patients which provide a forward input to central and frontal areas. As to the lack of occipital attentional effect in the sighted hemifield, one possibility is that given the presence of a blind hemifield, the attentional focus might be inevitably attracted to the sighted hemifield in both valid and invalid condition and therefore the visual input is overwhelming in the occipital areas. As a consequence, a differential attentional effect is less pronounced in early visual areas and more evident in the dorsal attentional system. As to hemispheric differences (contralateral versus ipsilateral) as a function of the visual hemifield stimulated, we did not find significant results in keeping with those of Gray et al. (2015) who found a bilateral occipital activity as a correlate of visual spatial attention and suggest that this depends on recruitment of neuronal populations from both hemispheres when attending only one hemifield. Thus, the contribution of the intact hemisphere of hemianopics is likely to have an important role as compensatory mechanism that maintains the ability to allocate spatial attention, even in absence of perceptual awareness.

The group analysis of the differential effect of attention (attended minus unattended) showed differences for the blind/left but not for the sighted/right hemifield. This difference did not involve the electrodes where the significant effect of attention was found in the blind field of patients. This might reflect, firstly, a similar effect of attention over extrastriate generators in the lesioned hemisphere and its counterpart in healthy participants, while the difference over the most anterior electrodes is likely due to the anatomical damage that reduces the capacity of the system to spread the activity forward. Secondly, the absence of difference over the ipsilateral hemisphere (i.e., intact hemisphere in patients) could be explained by a compensatory plastic mechanism following brain injury, e.g., enhanced interhemispheric interactions between the damaged and intact hemisphere (see Celeghin et al., 2017). These results provide important evidence that the neural mechanism of endogenous spatial attention can be at work independently from the presence of awareness as previously demonstrated by behavioral experiments (Kentridge et al., 1999, 2008). Thus, the main thrust of our study is to provide evidence that sustained attention to a blind hemifield triggers compensatory neural mechanisms that enhance the neurophysiological response but are not sufficient for perceptual awareness and this represents a kind of interesting dissociation between the two processes.

Putting together the results of our previous study (Sanchez-Lopez et al., 2017) in which we found reliable neural responses to visual stimuli presented to the blind hemifield and the present study, one obvious crucial question is what is missing for the emergence of perceptual awareness despite the presence of neural correlates of attention. One should consider that most of our hemianopic patients have large lesions including not only the primary visual cortex but also extrastriate areas and in some cases parietal and temporal areas. Moreover, three of them have clear evidence of optic radiation lesion. This complex picture of brain damage is obviously common to many hemianopic patients. A reasonable possibility is that a disruption of the interplay between striate/extrastriate visual cortex and parietal/frontal areas does not enable perceptual awareness to emerge because of a lack of top-down feedback. A broadly similar account has been proposed by Silvanto (2015). Interestingly, however, our present results show that the lack of the above mentioned interplay does not abolish the influence of attention on visual areas of the lesioned hemisphere even though this is not sufficient for perceptual awareness. At variance with the results of Kentridge and colleagues (Kentridge et al., 1999, 2004, 2008) our patients did not show unconscious behavioral evidence of an attention effect probably because their lesion was more extensive than the circumscribed visual cortex lesion of blindsight patient GY who was tested in the above mentioned studies. Of course, it would be important to test with SSVEP hemianopic patients with and without blindsight and with lesion strictly limited to V1.

A further related question is at what stage of central visual processing attention and awareness are dissociable. Important evidence comes from a magnetoencephalography (MEG) study by Wyart et al. (2012) who, with a metacontrast paradigm, found that at 100 ms from stimulus onset endogenous spatial attention enhanced early occipital MEG responses for both detected and undetected stimuli, and, therefore, was unrelated to conscious access and had no effect on stimulus detection. Thus, at an early stage, attention and awareness are dissociated and full perceptual awareness emerges later on when the two parallel independent processes cumulate their effects (see Tallon-Baudry, 2012). We believe that this picture is in accord with our present results.

In conclusion, we showed for the first time that the neural mechanisms of attention at early stages of visual processing are present independently from perceptual awareness. We believe that this result has relevance for constraining theories of the neural basis of awareness.

## Author Contributions

JS-L, SS, and CM contributed to the conception and design the study. JS-L and CP contributed to the data acquisition and organization of database. JS-L and SS performed the statistical analysis. JS-L and CM wrote the first draft of the manuscript. CP and NC wrote sections of the manuscript. All authors contributed to manuscript discussion, revision, reading, and finally approved the submitted version.

## Conflict of Interest Statement

The authors declare that the research was conducted in the absence of any commercial or financial relationships that could be construed as a potential conflict of interest.
